# Geographical clusters of dengue outbreak in Singapore during the *Covid*-19 nationwide lockdown of 2020

**DOI:** 10.1038/s41597-022-01666-y

**Published:** 2022-09-07

**Authors:** Liping Huang, Gaoxi Xiao, Hechang Chen, Xuetong Niu, Xiuju Fu, Haiyan Xu, George Xu, Stefan Ma, Janet Ong, Lee Ching Ng

**Affiliations:** 1 School of Electrical and Electronic Engineering, Nanyang Technological University, Singapore, Singapore; 2 School of Artificial Intelligence, Jilin University, Changchun, China; 3 Institute of High Performance Computing, Singapore, Singapore; 4 Ministry of Health, Singapore, Singapore; 5 Environmental Health Institute, National Environment Agency, Singapore, Singapore

**Keywords:** Viral infection, Environmental impact

## Abstract

Dengue, a mosquito-transmitted viral disease, has posed a public health challenge to Singaporean residents over the years. In 2020, Singapore experienced an unprecedented dengue outbreak. We collected a dataset of geographical dengue clusters reported by the National Environment Agency (NEA) from 15 February to 9 July in 2020, covering the nationwide lockdown associated with *Covid*-19 during the period from 7 April to 1 June. NEA regularly updates the dengue clusters during which an infected person may be tagged to one cluster based on the most probable infection location (residential apartment or workplace address), which is further matched to fine-grained spatial units with an average coverage of about 1.35 km^2^. Such dengue cluster dataset helps not only reveal the dengue transmission patterns, but also reflect the effects of lockdown on dengue spreading dynamics. The resulting data records are released in simple formats for easy access to facilitate studies on dengue epidemics.

## Abstract (summary)

**Table:** 

Measurement(s)	dengue infection locations
Technology Type(s)	NEA’s disclosured report
Factor Type(s)	Singapore’s dengue in in 2020

## Background & Summary

Dengue, a mosquito-borne viral infection transmitted to humans by *Aedes aegypti* (https://www.who.int/health-topics/dengue-and-severe-dengue), is prevalent in tropics and subtropics^1^. The dengue virus (DENV) is grouped into four closely related, but antigenically distinct and genetically diverse serotypes (DENV 1–4)^2^. Limited understanding of the immunological interactions among serotypes has hampered the development of effective vaccines^3^. Scientists estimate that dengue causes a worldwide symptomatic disease in 60 to 100 million population each year with 14 to 20 thousand annual deaths^4,5^.

As a city-state located in the tropics, Singapore’s climate and the highly urbanized environment make it ideal for the breeding of Aedes mosquitoes and transmission of dengue^6–8^. Dengue has been an endemic disease of Singapore and posing a public health challenge to the residents^9–11^. In 2020, Singapore had been facing the worst dengue outbreak. The cumulative number of dengue cases stood at more than 32000 as of 19 Oct 2020 (https://www.nea.gov.sg/dengue-zika/dengue/dengue-cases), which was the worst toll since 2013 when a total of 22,170 cases were reported (https://www.moh.gov.sg/docs/librariesprovider5/resources-statistics/reports/vector-borne–zoonotic-diseases.pdf). What makes the case of that year’s dengue spreading even more special is that the spike in early May coincided with Singapore’s COVID-19 lockdown period, named “circuit breaker (CB)”, from 7 April to 1 June. Before the lockdown, non-essential workers were no longer allowed to go to work on 27 March (https://www.gov.sg/article/covid-19-circuit-breaker-closure-of-workplace-premises). This nationwide lockdown was tightened from 7 April to 4 May when all business, social, or activity that cannot be conducted through telecommuting were suspended, and only essential services (e.g., supermarkets) remained open with minimum staff on premises. The lockdown was later extended to 1 June when limited additional economical activities were allowed (https://sso.agc.gov.sg/SL-Supp/S254-2020/Published/20200407?DocDate=20200407). The reopening process went through two different stages by the day of 9 July, 2020, where workplaces with safe management measures were reopened on 2 June (https://www.gov.sg/article/ending-circuit-breaker-phased-approach-to-resuming-activities-safely) and social gatherings of up to five people are allowed since June 19 (https://www.channelnewsasia.com/news/singapore/covid-19-phase-2-of-reopening-to-start-from-jun-19-social-12835758). Scientists argue that a factor that may have contributed to worsening the dengue outbreak that year was the lockdown measures: when more people were staying at home all the time, there may be more residential mosquito breeding and more opportunities for “blood meals” (https://www.channelnewsasia.com/news/singapore/mosquito-singapore-dengue-clusters-weekly-historical-high-12831856; https://edition.cnn.com/2020/07/03/asia/singapore-dengue-intl-hnk/index.html).

An open dengue dataset that covers the lockdown period may help facilitate exploring how the lockdown measures have affected the dengue outbreak. We collected the weekly dengue clusters from 15 February to 9 July in Singapore, which covers the whole nationwide lockdown period. A dengue cluster is formed and dynamically updated by NEA when two or more cases have onset within 14 days and are located within 150 m of each other based on the apartment block or the workplace address. To facilitate utilizing the dataset, we map the location record in the dengue clusters into the smallest spatial unit of Singapore, named the subzone (https://data.gov.sg/dataset/master-plan-2014-subzone-boundary-no-sea?resource_id=c30bfcc0-7e23-4959-b4d9-c5da5e00af54). Specifically, the Urban Redevelopment Authority (URA) of Singapore divides Singapore into 323 subzones, where each of them is typically centred around a focal point such as a neighbourhood centre or a commercial centre. Singapore covers a total area of 781.9 km^2^ and owns 5.7 million residents, with a high average population density at 7866 per km^2^ (https://www.citypopulation.de/php/singapore-admin.php). Except for those subzones with a population density lower than 10 per km^2^, the mean coverage of each of the other subzones is only about 1.35 km^2^. We map the dengue cases that are involved in the dengue clusters into the corresponding subzones.

After the map matching, each data record in the published dataset denotes an infection location (apartment block or workplace address) for a given week. A record contains record ID, latitude, longitude, date, cases, cluster label and the subzone ID. The date denotes the end of the week when the cluster was reported. The subzone ID is labelled by the shapefile that is described in the Methods section. With this dataset, both the spatial and temporal information of dengue transmission localities reported from 15 February to 9 July are recorded. This detailed record of an unprecedented dengue outbreak in Singapore before, during and after an unprecedented nationwide lockdown would be of high values to studies on risk estimation and system dynamics of dengue transmission, as well as the effects of lockdown on disease spreading and control.

## Methods

### Original data sources

The main data source is the dengue cluster records. Information of dengue clusters with infection locations comes from NEA (https://www.nea.gov.sg/dengue-zika/dengue/dengue-clusters). As aforementioned, a dengue cluster is formed by NEA when two or more cases have onset within 14 days and are located within 150 m of each other based the reported infection location. Here a location refers to the street address of an infected person’s workplace or homeplace down to the apartment block level. Such dengue cluster data is collected once or twice a week and each location is further labelled with the corresponding latitude and longitude by SGCharts (http://outbreak.sgcharts.com/data). The timestamp of the reported dengue clusters in the original data is recorded as the end date of the week within which the dengue case was reported. Note that the imported dengue cases are not included in the records as such cases are not disclosed by NEA.

Another open data source is the data that defines the subzone boundaries. The indictive polygon of the subzone boundary is defined by a shapefile, which is a data format widely used in the geographic information system (GIS). It can be downloaded from the online open data platform of Singapore (https://data.gov.sg/dataset/master-plan-2014-subzone-boundary-no-sea?resource_id=c30bfcc0-7e23-4959-b4d9-c5da5e00af54). For convenient usage, the shapefile data that defined the subzone boundary is also disclosed at figshare^12^. In the shapefile, each subzone boundary is defined by a polygon with a subzone ID.

### Mapping locations to subzones

**Fig. 1:**
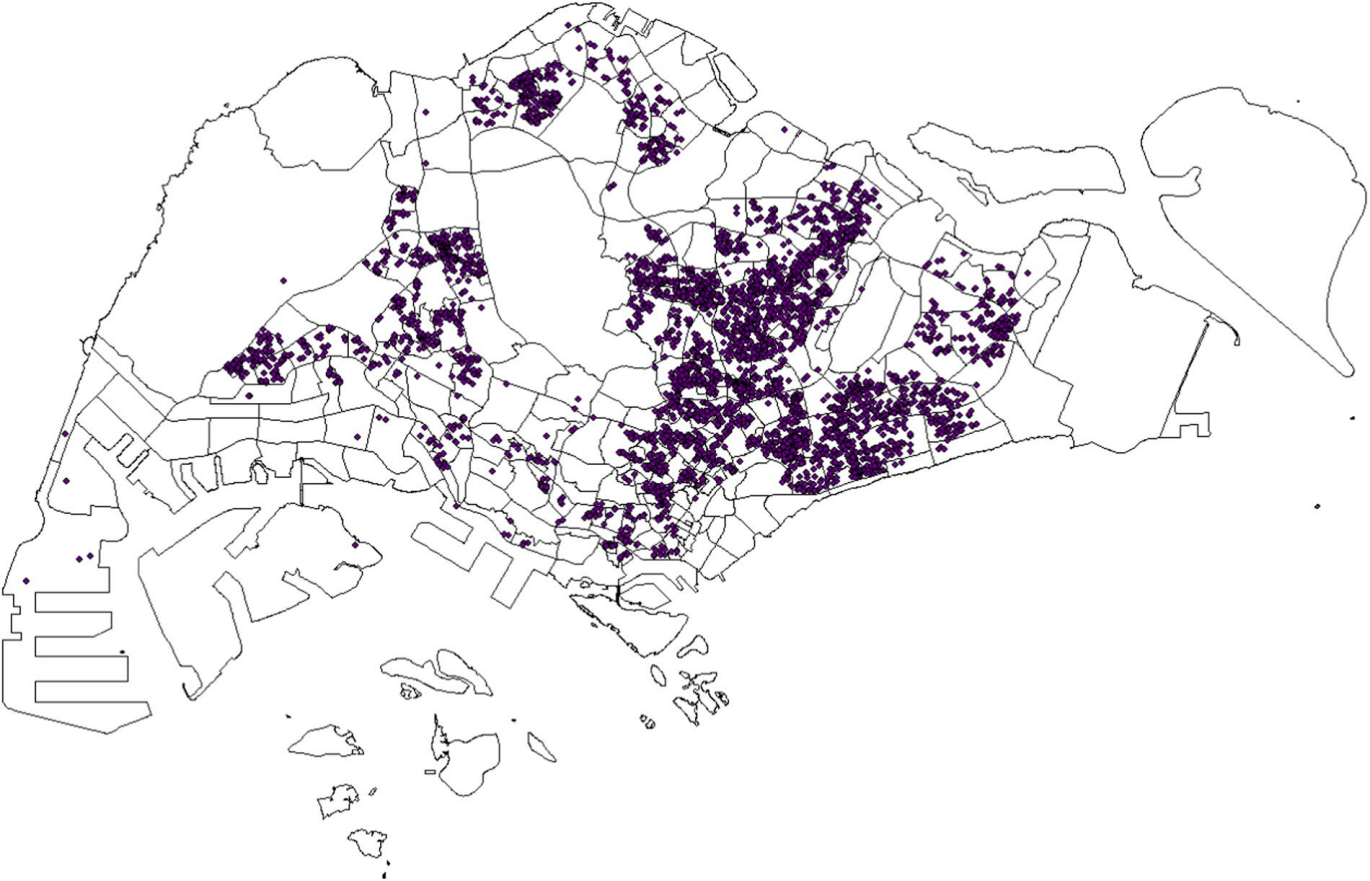
The geographical distribution of infecting locations and subzones.

**Fig. 2:**
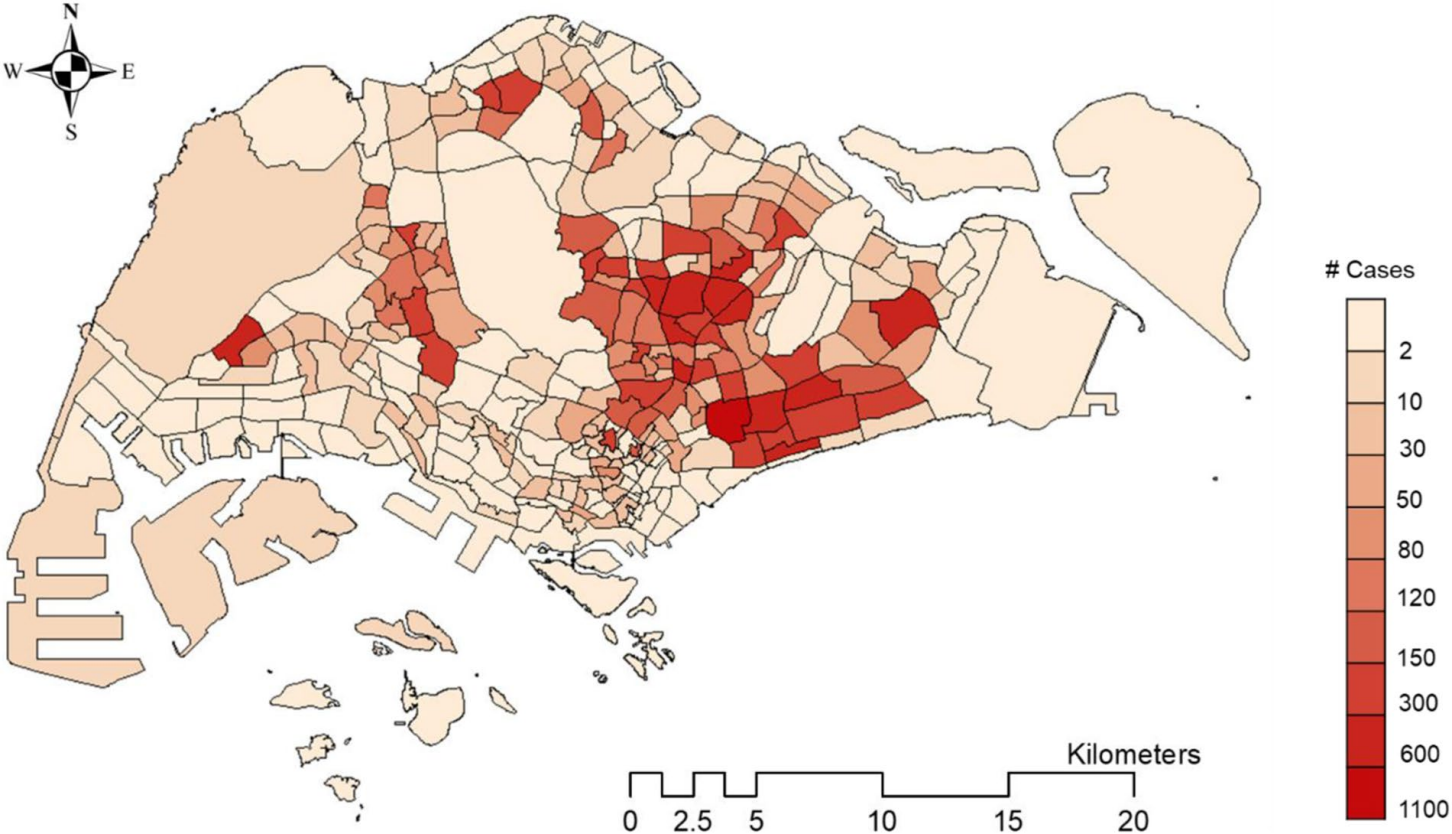
Spatial distribution of the infecting locations in subzones of Singapore from 15 February to 9 July. All infecting locations involved in the dengue clusters during this period are recorded and mapped to the corresponding subzones. The map is color-coded with the cumulative location number.

We utilize the ArcGIS Desktop platform with the ArcMap (version 10.4.1) module and the embedded ArcToolbox to match each location in the original dengue cluster data to the corresponding subzone. The downloaded shapefile that defines the subzone boundary is first added to ArcMap. The original location involved in the dengue clusters is stored in a comma-separated value (CSV) file, which contains the “latitude” and “longitude” of the reported infected location (apartment block or workplace address). The CSV file is then added to ArcMap, and the latitude and longitude are together displayed by choosing the “Geographic Coordinate Systems” as “WGS_1984”. After loading the CSV file, the data in the CSV file is uploaded into a software embedded table in the ArcMap. Before matching these locations to subzones, the embedded table should be converted to a shapefile by utilizing the toolbox of “ConversionTool- > To Shapefile”. Then we load the converted.shp file, which is opened as a table. The.shp file that defines the subzone boundary is then added to ArcMap. By using the “Spatial Join” toolbox to the two added layers, locations labelled with latitude and longitude are mapped to the corresponding subzones. With such map matching, the cumulative number of locations that are involved in the dengue clusters in each subzone during the period from 15 February to 9 July can be calculated. The geographical distribution of the mapping results is shown in Figs. 1 and 2.

## Data Records

The dataset is released as a CSV file, which can be openly downloaded^13^. The total time period that the dataset covers is from 15 February to 9 July in 2020. It contains a total of 16116 records. The data file is named “dengue outbreak_Singapore_2020.csv”, where each item (row) contains a few elements as follows:

- record ID: the data record id.
- latitude: numerical value for the latitude of the location.
- longitude: numerical value for the longitude of the location.
- date: the end date of the week when the location was reported to be enrolled in the dengue cluster by NEA. For example, “20200228” denotes the week from 22 February to 28 February in 2020.
- case number: number of reported dengue cases with onset in last 2 weeks at this location. New cases at this location for the corresponding week could be calculated by comparing the cases with those in the last week.
- cluster label: numerical label of a cluster for the given week.
- subzone ID: the ID of the subzone in which the workplace or homeplace of the case is located. Note that the subzone ID is defined by the shapefile that can be downloaded^12^. Each subzone is a spatial polygon as shown in Fig. 1.

The following Table 1 gives an example of the data items.

## Technical Validation

### Dengue infection locations in subzones over weeks

All dengue clusters are recorded in 21 weeks from 15 February to 9 July, which covers the nationwide lockdown period from 7 April to 1 June. The dataset of these 21 weeks is thus classified into three parts, namely before lockdown (BL), CB, and after lockdown (AL) respectively.

**Fig. 3:**
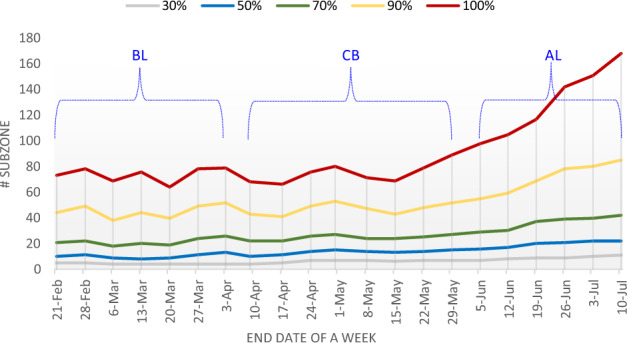
The number of top-ranked subzones that accumulatively contain at least 30%, 50%, 70%, 90%, and 100% locations involved in dengue clusters over the weeks.

For each week, we first rank the subzones in a descending order in terms of the number of locations inside the subzone in that week. Then we count the number of top-ranked subzones with cumulatively no fewer than a certain percentage of all the locations. The results are shown in Fig. 3. As can be observed, when the percentage is set to be 100%, meaning that all locations are taken into account, there was a significant increase in the subzone number starting in the second half of May. This indicates that the dengue transmission locations were dispersed to more subzones which were originally free of dengue clusters. It is interesting to observe that this dispersion started before lockdown officially came to an end. The reasons leading to it may need some further investigation.

Further observations show that, when the cumulative percentage is respectively set as 30%, 50%, and 70%, the corresponding subzone numbers remain relatively stable at low values. It means that, for most of time, a small number of subzones (fewer than 30 subzones before 19 June) contained no fewer than 70% of all the dengue infection locations. Even when the percentage value is set to be 90%, though the subzone number started to increase in May, it kept as being less than 85 in all weeks. Only when the percentage is set as 100%, a significantly larger number of subzones are enrolled starting in May. These newly enrolled subzones, combined together, contain no more than 10% of all the dengue infection locations. Take a specific week from 16 May to 22 May as an example, 90% of all locations were reported in 48 subzones, while the other 10% of locations are scattered in 31 subzones. Note that in the last two weeks of May, the number of the enrolled subzones increased while the number of reported infection locations still remained largely stable. When it came to June and July of 2020, however, the number of enrolled subzones further increased, accompanied with a significant increase in the number of infecting locations.

### Infection location number changes in subzones during lockdown

By looking into the time series of location number in each subzone, some details of the location number changes in the three periods (BL, CB, and AL respectively) could be observed. Specifically, given a subzone *z*, the average location number, *loc*_*i, j*_, in each period is calculated as

$${A}_{z}^{BL}=\frac{1}{{n}^{BL}}{\sum }_{i=z,j\in BL}lo{c}_{i,j}$$

$${A}_{z}^{CB}=\frac{1}{{n}^{CB}}{\sum }_{i=z,j\in CB}lo{c}_{i,j}$$

$${A}_{z}^{AL}=\frac{1}{{n}^{AL}}{\sum }_{i=z,j\in AL}lo{c}_{i,j}$$

where *i* is the subzone ID of the location record in the dataset and *j* is the end date of the week in the data record. *n*^*BL*^, *n*^*CB*^, and *n*^*AL*^ are respectively the number of weeks for each period (BL, CB, and AL). Looking into the time series, two different types of changes can be observed in different subzones, which are respectively defined as follows.

- CASE 1: the number of location number, which are involved in the dengue clusters, increased during the nationwide lockdown and it subsequently decreased after the lockdown.
- CASE 2: the location number sharply decreased during the lockdown and rose back after the lockdown.

We have that ${A}_{z}^{BL} < {A}_{z}^{CB} > {A}_{z}^{AL}$ for CASE 1 and ${A}_{z}^{BL} > {A}_{z}^{CB} < {A}_{z}^{AL}$ for CASE 2.

**Fig. 4:**
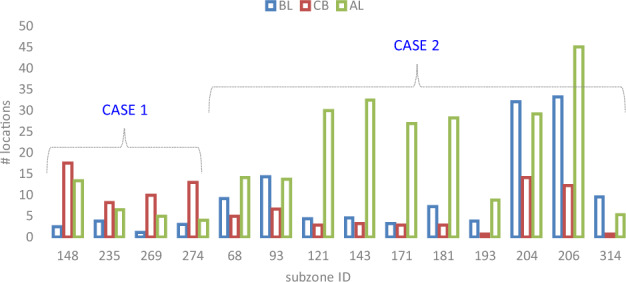
The average number of locations per week in subzones for CASE 1 and CASE 2, respectively. The first four subzones (with subzone IDs 148, 235, 269, and 274, respectively) belong to CASE 1, and the subsequent ten subzones belong to CASE 2.

**Fig. 5:**
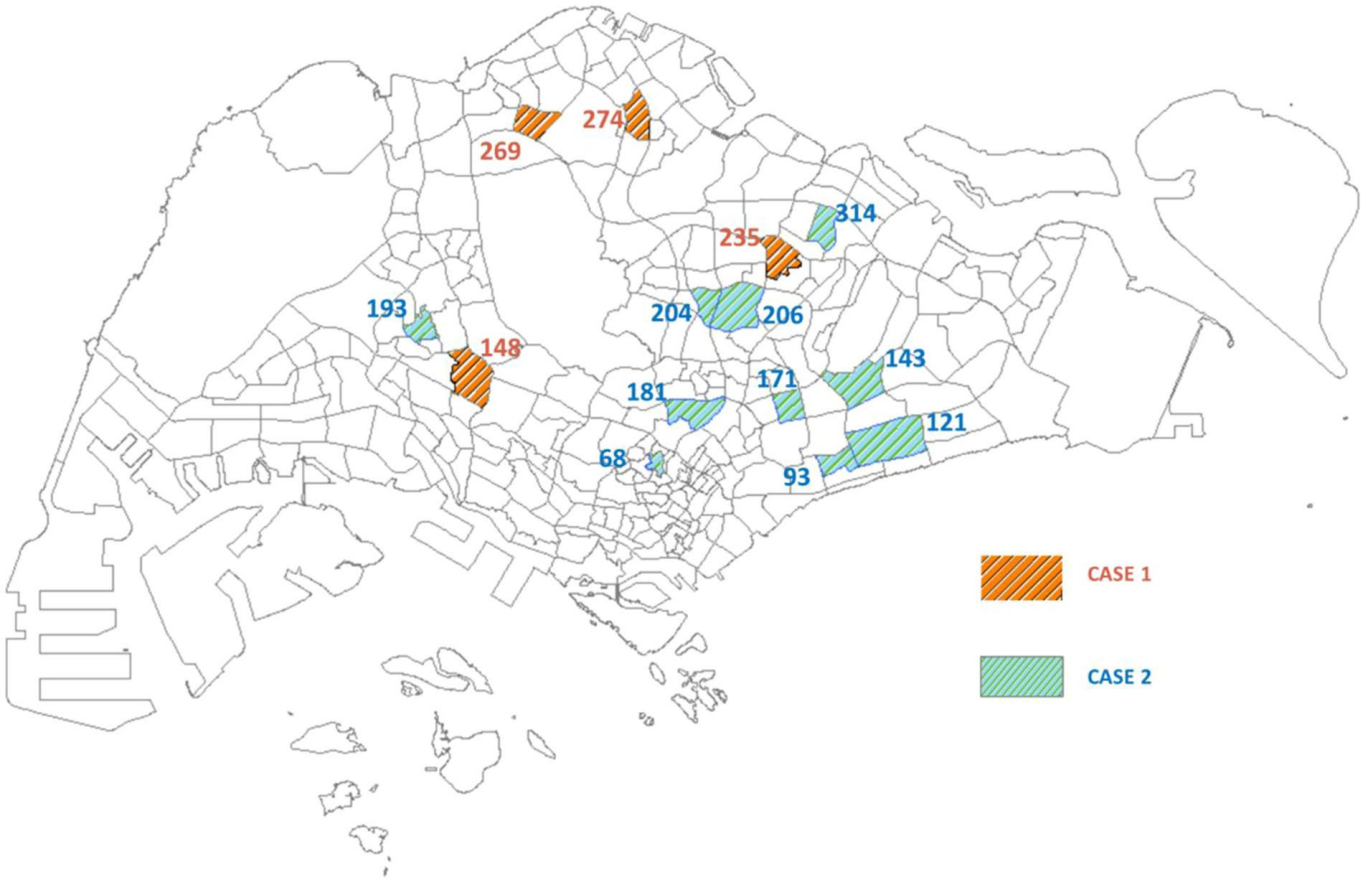
Geographical map of the identified subzones for CASE 1 and CASE 2, respectively. Four subzones are identified as belonging to CASE 1 and ten subzones for CASE 2. Both are labelled with the subzone IDs.

The time series of these two special cases may help anchor which areas in Singapore have been potentially influenced by the lockdown in terms of the reported location. The subzones for the two different cases are shown in Fig. 4. The corresponding geographical locations of these subzones for CASE 1 and CASE 2 are illustrated in Fig. 5.

## Usage Notes

The data are useful for investigating the spatial and temporal dengue risk at a fine-grained spatial resolution. An important note is that the dataset contains only the dengue cases that are involved in the dengue clusters. There are sporadic cases that are not part of clusters, and NEA presumes that such cases may acquire the disease elsewhere outside their home or workplace. Such sporadic cases are not involved in our dataset. Researchers who intend to model the dengue case variation may need to keep this fact in mind while using this data set. The dengue cluster data set may also be integrated with other datasets, such as the rainfall, temperature, and vegetation index data, etc., for estimating the dengue transmission risks.

The time period of this dataset covers the national lockdown period from April 7 to June 1 and involves seven weeks before the lockdown and six weeks after the lockdown as well. The commuting patterns started to change when lockdown preparation started on 27 March 2020 and experienced some drastic changes during the lockdown period before going through two different stages of reopening. As previous investigations have certified, human mobility is one of the main factors affecting the spatial transmission of dengue. Thus, this dataset may also contribute to helping assess the effects of human mobility on the spatial and temporal transmission of dengue in the highly urbanized city-state of Singapore.

An important factor that may need to be taken into account when using this data set to investigate the mobility effects on dengue transmission is the changes in population immunity: the main serotype of dengue virus in year 2020 was the DENV-3, whereas in the past three decades, the main serotype of dengue in Singapore had steadily been the DENV-1 or DENV-2. Another factor that needs to be considered is the resurgence of the mosquito population during this period of time. NEA reported observing a five-fold increase in the incidents of mosquito larvae in homes and common corridors in residential areas during the two-month circuit breaker period compared to those in two months prior.

**Table 1:** An example of the published data records.

record ID	latitude	longitude	date	case number	cluster label	subzone ID
1	1.387103	103.862363	200221	6	1	246
2	1.386371	103.861516	200221	5	1	246
3	1.387782	103.862708	200221	1	1	246
4	1.388717	103.861764	200221	5	1	246
5	1.386942	103.861363	200221	5	1	246

## Data Availability

Matlab codes for data analysis of calculating the dengue locations in subzones over weeks are available at the figshare repository^14^.
